# What every intensivist should know about intensive care unit admission criteria

**DOI:** 10.5935/0103-507X.20170073

**Published:** 2017

**Authors:** Seth T. White, Yenny R. Cardenas, Joseph L. Nates

**Affiliations:** 1 Critical Care Department, The University of Texas MD Anderson Cancer Center - Texas, United States.; 2 Critical Care Department, Fundación Santa Fé de Bogotá - Bogotá, Colombia.

## Introduction

The Institute of Medicine (IOM) published a landmark report in 2001^(1)^ recommending a thoughtful new health care delivery framework to improve the quality of care for the American population in the 21^st^ century. The IOM defined six quality domains that should be at the foundation of how we deliver critical care services: safety, timeliness, efficacy, efficiency, patient-centeredness, and equitability. However, critical care resources are limited and are not available to all, raising major concerns regarding how these resources are allocated. There are myriad reasons for the shortage of critical care services, which have led to the development of policies to improve the utilization of, and in turn often ration, these scarce resources.^(2)^

## Who needs to be in the intensive care unit?

The question of *who* requires intensive care support by a critical care team is complex. Critical care encompasses a broad variety of clinical conditions across different specialties and environments. The question of *how* to allocate critical care services is also not easy to answer; it depends upon many factors, which vary among individual institutions and include practitioner and resource availability among other considerations. It is easy to over-triage with minimal consequences if there is a surplus of beds available, but doing the same with a limited number of beds can have catastrophic repercussions for those later denied intensive care.

Recently, a task force of the Society of Critical Care Medicine (SCCM) published a new set of evidence-based guidelines to be used as a framework for enhancing and guiding clinical operations; the group provided updated intensive care unit (ICU) admission, triage and discharge strategies. These were aimed at not only helping practitioners but also guiding research in these particularly complex subject areas.^(3)^ The authors extensively evaluated all the relevant factors that play a role in these three processes, and through a systematic evidence-based approach highlighted the scarcity of evidence, which limited the strength of recommendations in most areas.

In regard to admission, there are several existing models to consider; the diagnosis model, the objective parameters model, and the prioritization model are the most commonly used. In the diagnosis model, practitioners use policies with lists of specific conditions that merit care in their respective units to guide admissions. This is an approach that involves listing a series of pathologies requiring care in the ICU. This approach is relatively straightforward (e.g., acute pulmonary edema) but is seldom used throughout Latin America. In the objective parameters model, specific thresholds are set whereby certain laboratory or physiologic parameters trigger evaluation and, when certain objective criteria are met, evaluation for admission occurs (e.g., sodium < 110 or > 170mEq/L). This is a more difficult approach to implement because there are not well-defined criteria for all systems. This approach is mostly used in combination with the diagnosis model and is often used as criteria for assessment by rapid response teams. In the prioritization model, patients are selected following a specially structured triage system prioritizing patients according to their needs and likelihood of benefiting from admission. In addition to the above models, others have proposed classifying patients by matching their hospital needs, rather than by the parameters of their illnesses. In this approach, patients are allocated to four different levels of care ranging from 0 to 3 (0 - ward, 1 - telemetry, 2 - intermediate medical unit, and 3 - ICU) based on their need for monitoring and/or interventions.^(4)^

Each of these approaches has its advantages and drawbacks, and none has been fully validated. The use of vital signs alone appears to be poorly specific and sensitive as a measure, precluding their use alone.^(5)^ More comprehensive scoring systems based on physiology and co-morbidity have been developed both in general^(6)^ and in specific subpopulations, such as hematopoietic transplant patients,^(7)^ to aid in admission decisions but have been validated only locally. Evidence for such scoring systems is poor, and the recommendation was made to not use a scoring system alone to determine eligibility for higher levels of care or discharge from ICU. Similarly, prognostic severity of illness scores are not recommended for use in making end of life decisions in individual patients.

## Making the decision to admit to intensive care

The decision to admit is multifaceted, encompassing many aspects of clinical practice. The initial criteria to be considered for admission are the need for an intervention that is not available elsewhere in the institution as well as clinical instability that places the patient at risk of dying or immediate deterioration.

To make recommendations on when to admit a patient, the SCCM task force created a list of the most important considerations, using aspects of the above models. The factors considered important by the task force included the identification of interventions that could be only provided in the ICU environment (e.g., life-supportive therapies), available trained personnel to care for the patient (including nursing and physician ratios), prioritization according to the patient's condition, clinical diagnosis, bed availability, objective parameters at the time of referral (e.g., elevated respiratory rate), potential to benefit from the interventions needed, and the patient's prognosis (Table 1). Also important are timing (minimizing delays in care) and ethical decision-making (avoiding discrimination and avoiding over- and under-triage). Patients' autonomy and wishes must be respected as central to any decision. Boarding (placing patients in beds not suited for their specialty) is associated with worse outcomes and should be avoided.

**Table 1:** Key factors to consider for intensive care unit admission

Patient	Facility	Principles of care
Patient agrees with ICU care and is:	Has:	Patient-centered
Critically ill	Clear admission policies	Equitable
Unstable	Operational protocols in place, such as a plan for surge capacity	Timely
Needs interventions that can only be provided in the ICU	Trained physicians	Efficient
Most likely will benefit from intensive care interventions	Trained nurses	Safe
	ICU beds available	Effective
	ICU equipment	Avoid providing nonbeneficial care in the ICU

ICU - intensive care unit.

## Triage

The existence of objective and pre-defined triage criteria is an essential component of disaster management plans, as recommended by the European Society of Intensive Care Medicine's (ESICM) Task Force on ICU triage during an Influenza Epidemic or Mass Disaster.^(8)^ Triage must endeavor to be as accurate as possible, but the SCCM recommends that over-triage is preferable to under-triage (that is, admitting patients to ICU who may not require it). Although slight over-triage may be preferred, significant over-triage may be deleterious if the service becomes overwhelmed, especially in times of crisis or extraordinarily high demand. Under-triage has been associated with increased mortality.^(3)^

A Task Force of the Council of the World Federation of Societies of Intensive and Critical Care Medicine (WFSICCM) has also commented on triage decisions for ICU admission.^(9)^ In their recent consensus statement, the group highlighted the role that (1) triage has in optimizing and making equitable critical care resources available, (2) the limitations of algorithms and protocols, (3) the importance of the collaborative intensivist approach in making the final decision to admit, and (4) the need for the efficient and organized use of resources at local and regional levels.

The SCCM's task force delineated a specific approach to prioritizing admissions during the triage process, proposing 5 levels. First priority is given to patients who are critically ill, can benefit from the intensive care/life support interventions in an ICU, and do not have limitations of care. Second priority is given to patients who are similar but have a questionable chance of benefiting from interventions because of advanced underlying diseases reducing their long-term survival or those who have specific limitations of care. Third priority is given to patients who are critical but can receive their needed therapies outside the ICU environment, such as non-invasive ventilation in an intermediate medical unit. Fourth priority is given to patients with priority three criteria but with lower chances of survival or with limitations of care. The fifth priority addresses patients with terminal conditions, who are moribund, or who would benefit from palliative care rather than inappropriately aggressive or heroic interventions.

The final category includes a controversial patient population for whom consensus has not been achieved. Nonbeneficial care provided in the ICU continues to occur, and there remains no universally accepted solution to this problem. The delivery of this (which some continue to inaccurately call *futile*) care in the ICU continues, and its potentially deleterious impact on others needing these resources remains unclear. Rationing has been undertaken in many countries with limited resources.

## Intensive care unit outreach/intensive care without walls

Because not all critically ill patients can be admitted to the ICU and some ICU admissions can potentially be prevented, many hospitals have recently implemented rapid response systems whereby clinicians trained in critical care assess patients who may be at risk of deteriorating on the ward and initiate suitable interventions. Rapid response systems have been shown to reduce ICU admissions and out-of-ICU cardiac arrests, though evidence for such programs has considerable methodological issues.^(10)^ The SCCM task force recommended using rapid response systems for the early review of acutely ill patients outside of the ICU to prevent unnecessary ICU admissions.

Critical care consult teams have been developed by several hospitals to aid in discharge and transfer to the ICU and assist in managing critically ill patients on the ward. As with rapid response systems, structures of such systems and their implementation vary widely and make detailed assessment difficult. Nonetheless, the SCCM recommends the use of critical care consult teams to help facilitate admissions and discharges from the ICU.

## Conclusion

The decision to admit or triage to the ICU is a complex everyday practice that can be overwhelming in times of high demand. We believe that by following a comprehensive approach based on the principles described above, any clinician can be capable of cutting-edge decisions. These principles are based on recommendations and strategies proposed by the IOM, SCCM, and consensus statements of the WFSICCM and ESICM. We must remember that ICU admission, discharge, and triage criteria are all continually evolving.

Regardless of the above guidance, intensivists must consider the local policies of the hospital and country in which they work. If such policies do not exist, it is essential to start by creating a detailed protocol in which everyone who takes care of critically ill patients knows when to call the ICU consulting team. This protocol would ensure that admission to, triage of and discharge from intensive care are transparent processes. These protocols must take into account the recommendations from various expert bodies that have indicated methods by which to optimize these processes. Once transfer to the ICU is agreed upon, it must be undertaken in a safe, efficient and timely manner to ensure high-quality patient care for all.
